# Dung Beetles, Dung Burial, and Plant Growth: Four Scarabaeoid Species and Sorghum

**DOI:** 10.3390/insects15121002

**Published:** 2024-12-18

**Authors:** Hasnae Hajji, Mariyem Rehali, Abdelkhaleq Fouzi Taybi, Jean-Pierre Lumaret, Youness Mabrouki

**Affiliations:** 1 Laboratoire de Biotechnologie, Conservation et Valorisation des Ressources Naturelles, Faculté des Sciences de Dhar El Mehraz, Université Sidi Mohamed Ben Abdellah, B.P. 1796 Fès-Atlas, Fez 30000, Morocco; hasnae.hajji@usmba.ac.ma (H.H.); younes_mab@hotmail.fr; 2 Laboratoire Biotechnologie Microbienne et Molécules Bioactives, Faculté des Sciences et Techniques de Fès, Université Sidi Mohamed Ben Abdellah, B.P. 1796 Fès-Atlas, Fez 30000, Morocco; mariyem.rehali@usmba.ac.ma; 3 Faculté Pluridisciplinaire de Nador, Équipe de Recherche en Biologie et Biotechnologie Appliquées, Université Mohammed Premier, Selouane, Nador 62700, Morocco; taybiaf@gmail.com; 4 Laboratoire de Zoogéographie, Université Paul Valéry Montpellier 3, Route de Mende, 34199 Montpellier, Cedex 5, France

**Keywords:** dung beetles, Scarabaeidae, sorghum growth, soil properties, regenerative agriculture, soil fertility, dung beetle diversity

## Abstract

This study examined the impact of dung beetles on both sorghum growth and the physico-chemical properties of the soil over a two-month period. Four dung beetle species (*Onthophagus vacca*, *Onthophagus marginalis* subsp. *andalusicus*, *Euonthophagus crocatus*, and *Gymnopleurus sturmi*) were introduced into experimental setups, consisting of containers filled with sterilised clay-loam soil, with three treatment groups: [cow dung + beetles], [cow dung only], and a control group (no dung nor beetles), in order to evaluate their effects on various growth parameters, including the plant height, biomass, leaf area, and chlorophyll concentration. Additionally, changes in soil properties were assessed, focusing on the organic matter content, pH, conductivity, enzyme activities (e.g., urease, phosphatase), and microbial load. The results revealed significant increases in all the measured parameters across the treatments involving dung beetles, emphasising their role in enhancing soil fertility and plant productivity. The study also highlights the variation in effectiveness among the species, suggesting that beetle diversity plays a crucial role in nutrient cycling. Integrating dung beetles into sustainable farming practices could, therefore, serve as a key strategy to promote resilient agriculture.

## Simple Summary

The burrowing activity of dung beetles improves soil fertility and hydrological properties and regulates biogeochemical cycles, influencing the growth and yield of agricultural crops, making them key species for environmental health and plant productivity. This study examines the impact of four dung beetle species on both sorghum growth and the physico-chemical properties of the soil over a two-month period. Changes in soil properties were assessed, focusing on the organic matter content, pH, conductivity, enzyme activities (e.g., urease, phosphatase), and microbial load. The results revealed significant increases in all the measured parameters across the treatments involving dung beetles, emphasising their role in enhancing soil fertility and plant productivity. The study also highlights differences in effectiveness among the species, suggesting that beetle diversity plays a crucial role in nutrient cycling. Integrating dung beetles into regenerative farming practices could serve as a key strategy to promote resilient agriculture.

## 1. Introduction

Dung beetles play a crucial role in both natural systems and in agroecosystems [1] by incorporating faecal matter into the soil, contributing to nutrient recycling. Their burrowing activity improves soil fertility and hydrological properties and regulates biogeochemical cycles, influencing the growth and yield of agricultural crops [2,3,4,5]. Integrating dung beetles into farming practices could improve soil quality and the availability of nutrients for plants, offering an ecological solution for agroecology. The best way to make this approach effective is to allow livestock to graze freely on fields used for seasonal crops after the harvest, leaving dung for the beetles to process.

Dung beetles relocate nutrients from the dung to the soil, increasing the plant food value and biomass [6,7]. Dung burial by dung beetles significantly increases the height of Japanese millet [8,9,10], the above-ground biomass of plants [6,11,12], the grain yield [10,12], and the plant protein content [13], while beetle activity significantly increases soil’s C, N, Na^+^, K^+^, Ca^2+^, Mg^2+^ and amino acid contents [14,15,16]. Galbiati et al. [9] also reported that the burial activity of dung beetles had positive effects on the diameter of maize cobs and the plant’s underground biomass.

Sorghum (*Sorghum bicolor* Moench L.) is grown in the Tangier, Tetouan, Chefchaouen, Taounate, Kenitra, Ben Slimane, and El Jadida Provinces in north-western Morocco. It is one of the major cereal crops in the north-western provinces, with large areas of land allocated to its cultivation and its use as a food crop [17]. Organic amendments (manure) help to improve yields [18], and often, after the harvest, the animals graze the residual stubble. This process shares similarities with the traditional cropping system Zaï in sub-Saharan West Africa, where planting pits (or *Zaï*) are used to optimize the retention of water and organic matter. This method, which restores soil fertility and supports resilient agriculture, highlights the potential role of organic amendments and soil biodiversity in sustainable agriculture [19,20].

In this context, we examined the contributions of four dung beetle species to plant growth and soil quality, *Gymnopleurus sturmi* (MacLeay, 1821); *Onthophagus vacca* (Linnaeus, 1767); *Onthophagus marginalis* subsp. *andalusicus* Waltl, 1835; and *Euonthophagus crocatus* (Mulsant & Godart, 1870) [Col. Scarabaeidae]. These species are abundant in Morocco, where they play a crucial role in recycling animal excrement.

In an experimental approach in a greenhouse, we studied the respective roles of dung and dung beetles on sorghum growth, measuring a range of plant growth parameters such as the lengths of plants, stems, and roots; the number of leaves; the plants’ aerial and root biomass; the surface area of leaves; the leaf water content; and the levels of chlorophyll a, b, and total chlorophyll, as well as carotenoids.

The hypothesis of this study was that dung incorporation into the soil by dung beetles should significantly increase soil nutrient availability and affect sorghum growth parameters. Complementing previous studies [8,12,14], this work aimed to enhance the understanding of the interactions between dung beetles and sorghum and to explore the potential implications of such interactions for sustainable agriculture in Morocco and elsewhere. The results could help optimize agricultural practices by better understanding the ecosystem services provided by dung beetles, while emphasising the importance of preserving soil biodiversity for more resilient and productive farming [19].

## 2. Materials and Methods

### 2.1. Study Site and Biological Material

As part of this study, a greenhouse experiment was conducted for two months at the Dhar El Mehraz Faculty of Science (FSDM) in Fez, Morocco. The substrate, characterised by a clay-loam texture, was collected near the town of Ifrane (33°32′ N–5°09′ W). The dung beetle species *O. vacca*, *O. m. andalusicus,* and *E. crocatus* were collected at Ifrane (33°32′ N–5°09′ W), and *G. sturmi* was collected at Fez-Sais (33°54′ N–4°59′ W). The plant tested in this experiment was sorghum (*Sorghum bicolor*), a fast-growing cereal grass widely cultivated for animal and human consumption. Sorghum is particularly adapted to arid and semi-arid conditions, making it an excellent plant for growth studies in the presence of dung beetles [21].

### 2.2. Soil Preparation and Experimental Set-Up

**Figure 1:**
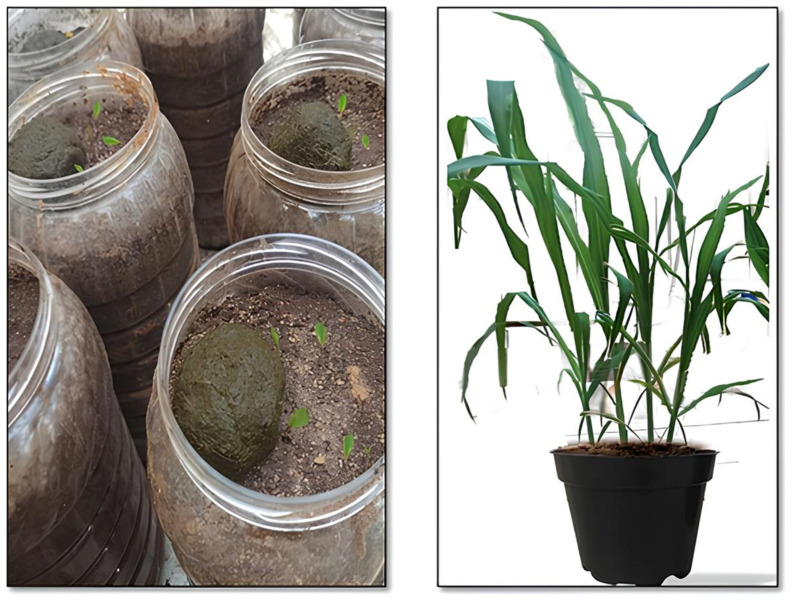
Growth of sorghum plants in pots. **Left**: the development of seedlings in soil with dung. **Right**: mature sorghum plants after two months.

The soil was sieved and sterilised to eliminate coarse particles and undesirable microorganisms. The initial pH of the soil was 6.48. The soil was then redistributed into eighteen circular plastic containers, each 33 cm in diameter and 35 cm deep. The containers were subdivided into three groups: [cow dung + beetles], [cow dung only], and control [no dung nor dung beetles], with three replicates per group. Each container had four sorghum plants, equally spaced. Seeds were sown to a depth of 2 cm and watered immediately after planting to ensure good germination (Figure 1). However, due to potential seedling mortality, the final number of growing plants could vary. The dung beetles were introduced into the containers one week before sowing the seeds to allow them time to acclimatize.

In the [cow dung + beetle] group, the containers received 100 g of fresh cow dung on the surface, renewed every three days, with ten individuals of each species (five males and five females) per container. The [cow dung only] group received 100 g of dung, renewed every three days. The control group received neither dung nor beetles. Unused dung was removed and replaced with fresh dung throughout the two-month experimental period.

### 2.3. Measurements of Physico-Chemical Parameters and Plant Growth

During the experiment, each replicate was watered every three days until the soil reached its field capacity. The field capacity was determined by saturating the soil with a known volume of water and allowing any excess to drain. The volume of water recovered was then measured, and the field capacity was calculated as the difference between the volume of water applied and the volume of water recovered. Plant and soil measurements were carried out after 60 days, before the plants had reproduced. The height of each plant was measured to assess stem growth. The aerial parts (shoots) and roots were weighed separately after the harvest to assess the total mass of the plant and its distribution into the roots and shoots. The fresh weight was measured immediately after harvesting, then the dry weight was measured after oven drying (natural convection oven, Memmert, Type U 15) at 80 °C for 2 to 3 days. The total number of leaves on each sorghum plant was counted to assess leaf density and leaf production. The leaf area was measured using Mesurim Pro software, version 3.4 [22]. Leaf images were captured using the CamScanner application on a cell phone (version 6.75.0.2411050000, INTSIG Information Co., Ltd., Singapore). This application automatically adjusts the sharpness and brightness to obtain high-quality images. Three leaves per plant were captured, with four plants per container, totalling twelve leaves per replicate. The resulting images were then imported into Mesurim Pro v 3.4 (Fr), where we used the automatic contouring tool to measure leaf area.

### 2.4. Chlorophyll and Carotenoids Measurements

The chlorophyll concentrations in leaves (a, b, and total) were quantified using the method described by Arnon [23]. Leaves were harvested and shredded in acetone in darkness to extract the chlorophyll. The acetone solution containing the chlorophyll was then centrifuged at 5000 rpm for 10 min (Centrofriger-BLT, Selecta S.A., Abrera, Spain) to separate the chlorophyll pigments from the other cellular components. The chlorophyll concentrations were determined by measuring the absorbance of the solution at different wavelengths by using a UV-2005 spectrophotometer (Selecta, model 4120020, Abrera, Spain). This method enables the chlorophyll contents of the leaves to be accurately assessed, providing information on the photosynthesis capacity of the plants under the different experimental conditions.

### 2.5. Leaf Water Content

To determine the leaf water content, the fresh mass (fm1) and dry mass (dm2) of the leaves after drying at 100 °C for 2 h were compared. Three leaves per plant, similar in size, were selected and weighed with an APX-200 balance (Denver Instrument GmbH, Göttingen, Germany) (0.01 mg precision). Three measurements were taken per leaf. The water content (%) was determined by using the equation [fm1 − dm2]/fm1 × 100.

### 2.6. Soil Physico-Chemical Properties

The pH was measured with a Thermo Scientific™ Orion Star™ A324 portable pH/ISE multimeter (Thermo Fisher Scientific, Waltham, MA, USA). Electrical conductivity (expressed in microSiemens per meter μS/m) was measured using a Bante 540 portable conductivity meter (Bante Instruments Inc., Sugar Land, TX, USA), which offers a measurement range of 0 to 200 mS/cm, with ± 0.5% accuracy. The soil organic matter content was determined by calcination after drying at 105 °C for 24 h in a Carbolite muffle furnace. For each experimental condition, a 5 g soil sample was analysed, and each measurement was repeated three times.

### 2.7. Enzymatic Activities

Soil enzyme activities were analysed to assess biogeochemical cycles, in particular β-galactosidase for the carbon cycle, alkaline phosphatase for the phosphorus cycle, and urease for the nitrogen cycle.

The soil samples were oven-dried at 105 °C for 24 h before the analysis. For each treatment, 5 g soil samples were used for the enzymatic measurements. The measurements were conducted in triplicate to ensure consistency and reliability; the values were plotted in each graph, the error bars representing the standard deviation (*sd*) of the three measurements.

The method described by Hoffmann and Teicher [24], which was employed to measure β-galactosidase activity, involved the incubation of the soil with a specific substrate and the measurement of the reaction product spectrophotometrically. Alkaline phosphatase activity was quantified by the colorimetric estimation of the p-nitrophenol released after the soil incubation with a buffered p-nitrophenyl sodium phosphate solution (pH 6.5) and toluene at 37 °C for one hour, in accordance with the method by Tabatabai and Bremner [25]. Urease activity was determined by measuring ammonium release after the incubation of the soil sample with trimethylamine, urea solution, and toluene (0.2 mL) at 37 °C for two hours, according to the method defined by Zantua and Bremner [26].

### 2.8. Microbial Load

Soil samples were collected at various depths using sterile tools and stored in sterile containers. Ten grams of each soil sample was added to 90 mL of sterile water to create a 10^−1^ dilution. Serial dilutions were prepared by transferring 1 mL of the initial dilution into 9 mL of sterile water, repeating this process to achieve the desired dilution levels, ranging from 10^−2^ to 10^−5^. A drop of the final solution was spread on nutrient agar (NA) gel for bacteria, on a potato dextrose agar (PDA) gel with 50 mg/L chloramphenicol for fungi, and on an actinomycete isolation agar (AIA) gel for actinomycetes. The plates were incubated at 30 °C for 24–48 h for bacteria, at 25 °C for 3–5 days for fungi, and at 28 °C for 5–7 days for actinomycetes. Colonies were counted after incubation and the colony-forming units (CFU) per gram of soil were calculated using standard methodologies [27,28].

### 2.9. Statistical Analysis

The statistical analysis was performed using R software (version 4.1.3) [29]. When the conditions of normality and/or homoscedasticity were fulfilled, an ANOVA was performed, followed by Tukey tests. Conversely, when these conditions were not satisfied, a Kruskal–Wallis test was used, followed by Wilcoxon tests [29]. The statistical significance level was set at the 0.05 probability level.

## 3. Results

### 3.1. Agronomic Parameters

**Figure 2:**
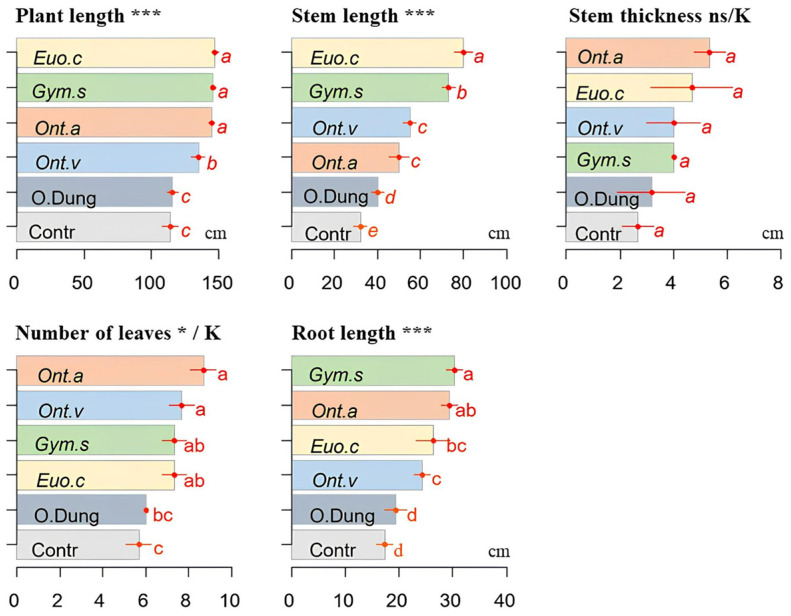
Effects of four dung beetle species (*E. crocatus* (*Euo.c*), *G. sturmi* (*Gym.s*), *O. m. andalusicus* (*Ont.a*), and *O vacca* (*Ont.v*)) combined with dung on sorghum growth, compared with dung alone, (O. Dung), no dung, or no dung beetle control (Contr). Means with the same letters are not significantly different. Error bars represent the standard deviation (*sd*) of the observations. *p*-values: * *p* ≤ 0.05; *** *p* ≤ 0.001. ns: not significant. A “K” above the figure indicates that the non-parametric Kruskal–Wallis test was used, while figures without this label correspond to the data analysed using ANOVA.

The different dung beetle species had varying effects on plant growth (Figure 2). The presence of all the species significantly increased the plant length and the stem length, compared with the control (*p* < 0.001). *Onthophagus m. andalusicus* and *O. vacca* had a significant effect on the number of leaves (*p* < 0.05), while *G. sturmi* had a significant effect on the root length (*p* < 0.001). However, none of the species showed a significant effect on the stem thickness (Figure 2).

**Figure 3:**
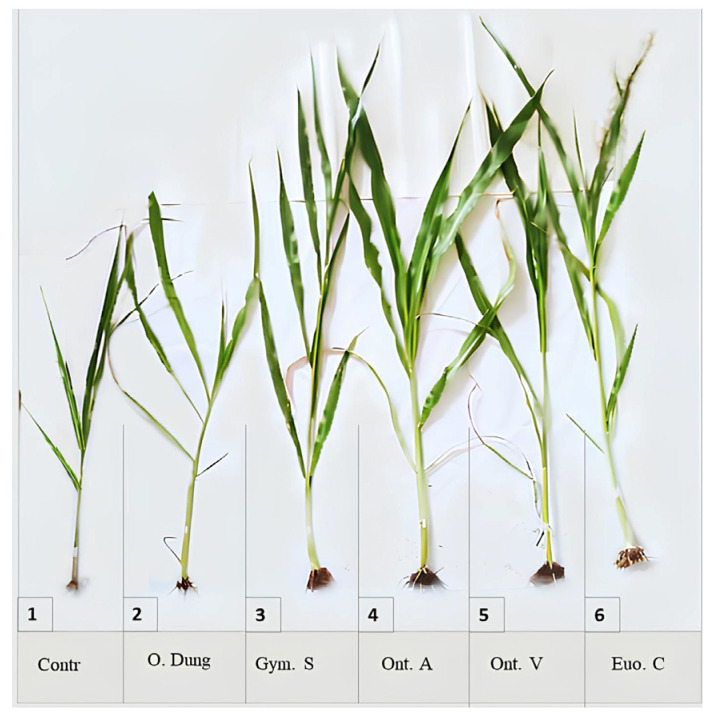
The effects of different treatments on the shoots of sorghum plants. 1: control; 2: only dung; 3: *G. sturmi*; 4: *O. m. andalusicus*; 5: *O. vacca*; 6: *E. crocatus*. Note: The root systems have been removed to highlight the aerial parts of the plants.

The containers with only dung also showed a significant improvement in the stem length compared with the control (no dung nor dung beetles) (*p* < 0.05), although this effect was less marked than that observed with dung beetles. However, as with the dung beetles, dung alone had no significant effect on the stem thickness (*p* > 0.05) (Figure 3).

### 3.2. Biomass of Plants

**Figure 4:**
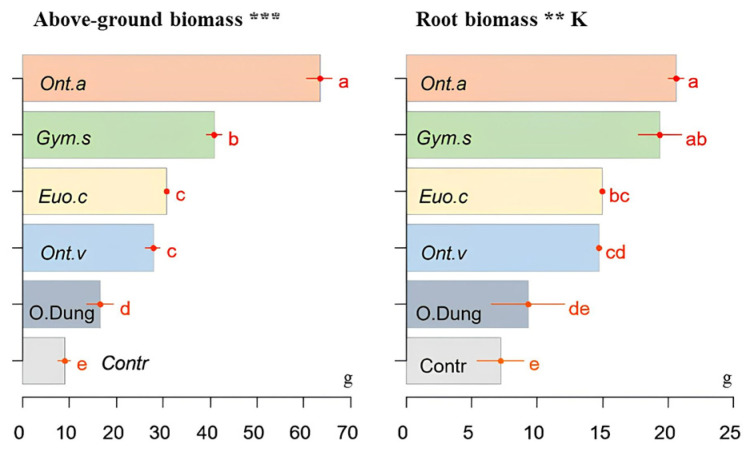
The effects of four dung beetle species (*E. crocatus* (*Euo.c*), *G. sturmi* (*Gym.s*), *O. m. andalusicus* (*Ont.a*), and *O. vacca* (*Ont.v*)) combined with dung on the biomass (in g) of the aerial and root parts of sorghum plants, compared with the [only dung] group (O.Dung) and the control group [no dung nor dung beetle] (Contr). Means with the same letters are not significantly different. Error bars represent the standard deviation (*sd*) of the observations. *p*-values: ** *p* ≤ 0.01; *** *p* ≤ 0.001. A “K” above the figure indicates that the non-parametric Kruskal–Wallis test was used, while figures without this label correspond to data analysed using ANOVA.

Figure 4 shows the effects of dung beetles on the biomass of the above-ground and root parts of sorghum plants, compared with the group [cow dung only] and the control group [no dung beetles nor dung]. The species *O. m. andalusicus*, *G. sturmi*, and *E. crocatus* had a significant effect on the aerial biomass (*p* ≤ 0.001) and root parts of the plants (*p* ≤ 0.01). *Onthophagus vacca* also caused significant effects, but to a lesser extent. The plants in the [cow dung only] group had a higher biomass than the plants in the control group [no dung beetles nor dung].

### 3.3. Leaf Area and Water Content

**Figure 5:**
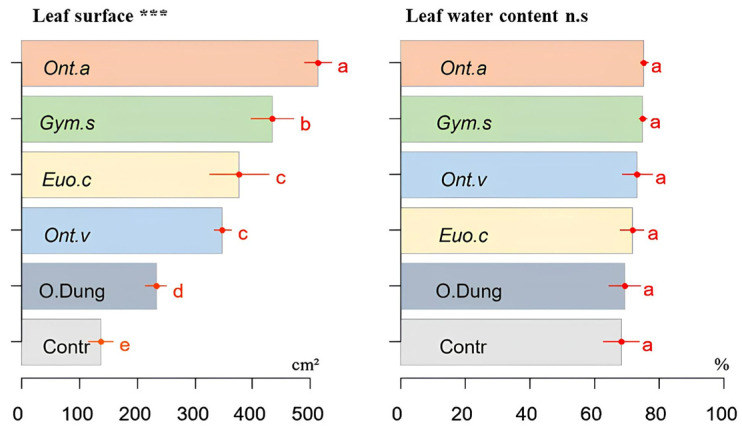
Th effects of the activities of four dung beetle species (*E. crocatus* (*Euo.c*), *G. sturmi* (*Gym.s*), *O. m. andalusicus* (*Ont.a*), and *O. vacca* (*Ont.v*)) on the leaf surface area (in cm^2^) and leaf water content (as %) of sorghum plants, compared with the [only dung] group (O.Dung,) and the control group [no dung nor dung beetles] (Contr). Means with the same letters are not significantly different. Error bars represent the standard deviation (*sd*) of the observations. *p*-values: *** *p* ≤ 0.001. n.s: not significant.

The burrowing activity of all the dung beetle species contributed to a significant increase (*p* ≤ 0.001) in the plant leaf area compared with the two other groups [cow dung only] and the control (Figure 5), but without any change in the water content of the leaves compared with these groups.

### 3.4. pH, Conductivity, and Soil Organic Matter

**Figure 6:**
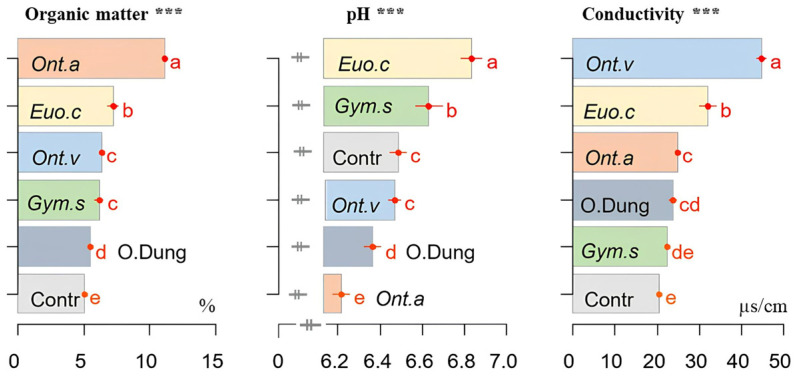
The effects of the activities of four dung beetle species (*E. crocatus* (*Euo.c*), *G. sturmi* (*Gym.s*), *O. m. andalusicus* (*Ont.a*), and *O. vacca (Ont.v*)) on the organic matter level in soil (%), the soil pH, and the soil conductivity (in microSiemens per cm; μS/cm) compared with the [only dung] group (O.Dung) and the control group [no dung nor dung beetles] (Contr). Means with the same letters are not significantly different. Error bars represent the standard deviation (*sd*) of the observations. *p*-values: *** *p* ≤ 0.001.

The action of dung beetles resulted in a significant increase in the organic matter content in the experimental soil, with the differences depending on the species, with *O. m. andalusicus* being the most effective species (Figure 6).

The soil conductivity differed also, according to the species activity. The values were significantly higher (*p* ≤ 0.001) with *O. vacca*, followed by *E. crocatus* and *O. m. andalusicus*. The values for *G. sturmi*, on the other hand, did not differ significantly from the [cow dung only] group and the control group (Figure 6).

### 3.5. Chlorophylls and Carotenoids

**Figure 7:**
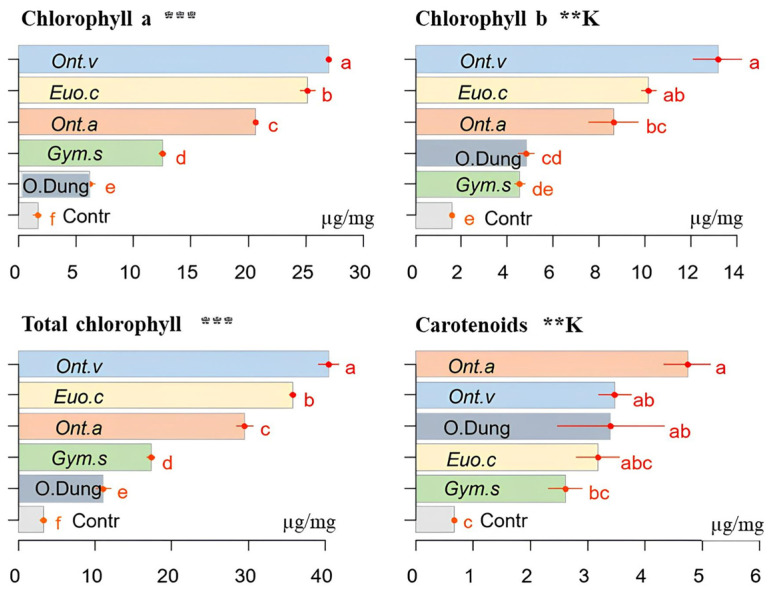
The effects of the activities of four dung beetle species (*E. crocatus* (*Euo.c*), *G. sturmi* (*Gym.s*), *O. m. andalusicus* (*Ont.a*), and *O. vacca* (*Ont.v*)) on the foliar pigments of sorghum plants (in μg/mg) compared with the [dung only] group (O.Dung) and the control group [no dung nor dung beetles] (Contr). Means with the same letters are not significantly different. Error bars represent the standard deviation (*sd*) of the observations. *p*-values: ** *p* ≤ 0.01; *** *p* ≤ 0.001. A “K” above the figure indicates that the non-parametric Kruskal–Wallis test was used, while figures without this label correspond to the data analysed using ANOVA.

The burial of the dung in the soil by dung beetles resulted in significantly higher levels of chlorophyll a and b, total chlorophyll, and carotenoids in the sorghum plants compared with both the [only dung] group and the control group [no dung nor beetles], but with differences between the species (Figure 7). The activity of *O. vacca* significantly increased the concentration of chlorophyll a (*p* ≤ 0.001) and b (*p* ≤ 0.01), as well as the total chlorophyll (*p* ≤ 0.001) in the leaves; the action of *G. sturmi* was the least marked compared with the other species.

Figure 7 shows that the carotenoid levels in the sorghum leaves differed only slightly both between the treatments and between the species. The letters on the bars of the graphs (ab, abc, bc) illustrate the statistical differences observed. The only noticeable exceptions were *E. crocatus* and *O. vacca*, whose burrowing activity resulted in higher levels of carotenoids compared with the control [no dung], while the activity of the other species resulted in little difference compared with other treatments.

### 3.6. Enzymatic Activity and Microbial Load

**Figure 8:**
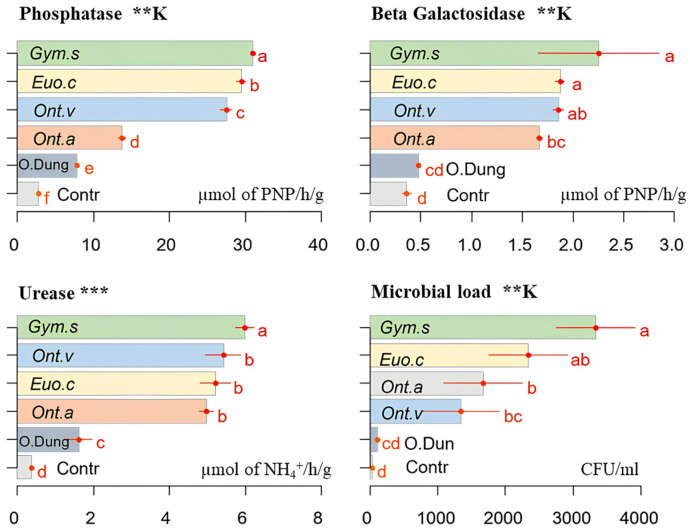
The effects of the activities of four dung beetle species (*E. crocatus* (*Euo.c*), *G. sturmi* (*Gym.s*), *O. m. andalusicus* (*Ont.a*), and *O. vacca* (*Ont.v*)) on the soil enzyme activity and the microbial load compared with the [only dung] group (O.Dung) and the control group [no dung nor dung beetles] (Contr). Phosphatase and beta-galactosidase concentrations are expressed in the μmol of *p*-nitrophenol (PNP) produced per hour and per gram of soil. Urease is expressed in the μmol of NH4+ produced per hour and per gram of soil. The microbial load is expressed in CFU/mL (colony-forming units per mL). Means with the same letters are not significantly different. Error bars represent the standard deviation (*sd*) of the observations. *p*-values: ** *p* ≤ 0.01; *** *p* ≤ 0.001. A “K” above the figure indicates that the non-parametric Kruskal–Wallis test was used, while figures without this label correspond to the data analysed using ANOVA.

Dung beetles increased both the enzyme activity and the soil microbial load, compared with the [cow dung only] group and the control [no dung nor beetles]. *Gymnopleurus sturmi* had the highest significant effect on phosphatase levels (*p* ≤ 0.01), followed by *E. crocatus*, *O. vacca,* and *O. m. andalusicus*. The lowest values were obtained with the other groups, which were without beetles or dung (Figure 8).

Similarly, the activity of all the dung beetle species significantly increased beta-galactosidase levels (*p* ≤ 0.01) in the same order as with phosphatase (*G. sturmi* and *E. crocatus* showed the highest effects). The lowest levels of both beta-galactosidase and phosphatase activity were recorded with the treatments [cow dung only] and control, underscoring the positive impact of dung beetles on enzyme activity. Figure 8 illustrates this trend, with clear differences in the enzyme levels across the various treatments. Additionally, all the dung beetle species significantly increased (*p* ≤ 0.001) the urease concentrations in the soil, with the lowest values observed in the other treatments. The microbial load was increased by the activity of all the species compared with the other treatments, most notably by *G. sturmi* and *E. crocatus*.

## 4. Discussion and Conclusions

The present study demonstrated that the burial and processing of dung by four dung beetle species had a significant impact on both the growth of sorghum plants and the soil properties. All the species had positive effects on the plant length, stem, and root length, as well as the leaf number, compared with the treatment [cow dung only] and the control [no dung nor dung beetles].

Livestock dung is a rich source of minerals, bacteria, and microbial enzymes [30] and enriches the mineral and organic matter content of the soil. In the absence of dung beetles, dung remains on the surface and these resources are largely unused. Dung beetle burial accelerates dung decomposition and enhances bioturbation, releasing nutrients into the soil [4]. This process improves nutrient mobilisation, resulting in an increased plant biomass [10,31,32,33], with effects comparable, to some extent, to those of chemical fertilisers [34]. Our results showed a significant increase in the sorghum biomass, in agreement with studies by Bornemissza and Williams [8], who observed an increase in the plant height and the aerial biomass in a comparable context.

Containers with only dung deposited on the surface were used as the reference point to assess the impact of dung beetles on the plant growth and soil physico-chemical parameters. Although dung by itself improved some of the growth parameters and soil properties, the addition of dung beetles considerably amplified these effects. The plants were significantly taller in the presence of the dung beetles compared to those grown with dung only deposited on the surface. This can be attributed to the fact that dung alone on the surface releases its nutrients slowly, whereas the combined action of dung with dung beetles results in a more rapid relocation of nutrients. These results are consistent with previous studies, which demonstrated that the increased nitrogen mobilisation facilitated by the dung beetles improved cell division, thereby boosting vegetative growth [35,36].

*Onthophagus m. andalusicus* and *G. sturmi* were particularly effective in improving the plant biomass, both above and below ground. These results support the findings of Bang et al. [6] and Brown et al. [37] who reported that dung beetles improved the structure and hydrological properties of soil, leading to better plant growth. The leaf surface was positively influenced by the presence of dung beetles, with the strongest effect being observed in the presence of *O. m. andalusicus* and *G. sturmi*. These species increase the phosphorus and nitrogen concentrations in the soil to a depth that makes these nutrients immediately available to plants [14]. The water content of plant tissues was not affected by the addition of dung beetles, probably because the plants were watered every three days.

Chlorophyll a and b and the total chlorophyll levels, as well as carotenoids, increased with the addition of dung beetles. The activities of *O. vacca* and *E. crocatus* resulted in significant increases in chlorophyll levels, which is consistent with the effects of dung beetles on Chinese cabbage, where dung beetles increased the enzymatic activity and bacterial diversity in the soil, leading to greater photosynthesis and plant growth [38]. These results show that dung beetles, by facilitating the release of nutrients from dung when mixed with soil particles, increase photosynthesis and, thus, contribute to better plant growth. Also, soil fertilisation with dung can play an important role in increasing the chlorophyll content by providing a fraction of Mg^2+^ ions [39].

Dung beetles, by facilitating the decomposition of organic matter in the soil, improve the availability of nutrients for plants [40]. Soil enzyme activity is recognised as a key indicator of microbial function for the conservation of nutrients related to soil fertility and health [41]. Our study showed a significant increase in the soil microbial load and enzymatic activities, namely those of acid phosphatase, urease, and β-galactosidase, with the addition of dung beetles. This pattern is consistent with the results of Menéndez et al. [42] and Slade et al. [5], who demonstrated that dung beetles increased bacterial diversity and microbial respiration, thus contributing to soil fertility. The results of Kaleri et al. [38] supported these results by showing that dung beetles significantly increased the activity of soil enzymes such as acid phosphatase, urease, and β-glucosidase. The activity of the beetles on the soil pH was more varied, with a higher acidification of the soil by *O. m. andalusicus*, possibly due to the mineralisation of the greater quantity of organic matter buried by this species, at a greater depth in the soil [14].

β-galactosidase is essential to the process of organic matter mineralisation because it catalyses the decomposition of polysaccharides in the detritus and transforms them into available carbon [43]. Urease activity is also a very reliable enzymatic indicator of the mineralisation of organic nitrogen in soil [44]. Phosphatases play an important role in the solubilisation of phosphates, making them available for uptake by plants [45]. Our results showed that the activity level of these enzymes was significantly increased by the addition of dung beetles, which enhanced the availability of nutrients for plants and, consequently, their growth.

Dung burial by *Digitonthophagus gazella* (Fabricius) [Scarabaeidae] improves the soil structure by increasing the nitrogen and phosphorus content in the soil, which promotes the better growth of crops such as *Brachiaria decumbens* Stapf. [Poaceae] [34]. Our results corroborate those from an earlier study, in which we tested the effects of these same four dung beetle species on soil fertilisation. In that study, we demonstrated that they significantly increased the level of nutrients in the soil, such as nitrogen, potassium, and phosphorus [14]. The activity of dung beetles (bioturbation, aeration) also has positive effects on the general health of the soil, particularly through an increase in enzyme activity, thus contributing to better plant growth, as demonstrated in the present study.

The integration of dung beetles into agricultural systems has beneficial effects on plant growth, particularly in regions with nutrient-poor soils. Studies by Bornemissza and Williams {8] on Japanese millet and by Rougon and Rougon-Chassary [16] on pearl millet in Niger confirmed that dung buried by dung beetles improved productivity in poor sandy soils. In Niger, traditionally, the breeders brought their livestock to the harvested fields, thus fertilizing the agricultural field before the next harvest [12]. The “downside” of this practice is that a portion of the carbon and nutrients are being exported from the harvested fields in the grazing animals.

## Data Availability

The data presented in this study are available on request from the first author (H.H.); they form part of her PhD thesis.
